# Sensory processing abilities of children with ADHD

**DOI:** 10.1590/bjpt-rbf.2014.0043

**Published:** 2014

**Authors:** Vitoria T. Shimizu, Orlando F. A. Bueno, Mônica C. Miranda

**Affiliations:** 1 Departamento de Educação, Universidade Federal de São Paulo (UNIFESP), São Paulo, SP, Brazil; 2 Departamento de Psicobiologia, Escola Paulista de Medicina (EPM), UNIFESP, São Paulo, SP, Brazil

**Keywords:** ADHD, sensory processing, sensory profile, learning, behaviour, rehabilitation

## Abstract

**OBJECTIVE:**

To assess and compare the sensory processing abilities of children with Attention Deficit/Hyperactivity Disorder (ADHD) and children without disabilities, and to analyze the relationship between sensory processing difficulties and behavioural symptoms presented by children with ADHD.

**METHOD:**

: Thirty-seven children with ADHD were compared with thirty-seven controls using a translated and adapted version of the "Sensory Profile" answered by the parents/caregivers. For the ADHD group, Sensory Profile scores were correlated to behavioural symptoms assessed using the Child Behaviour Check List (CBCL) and the Behavioural Teacher Rating Scale (EACI-P). The statistical analyses were conducted using the Mann Whitney test and Pearson correlation coefficients.

**RESULTS:**

: Children with ADHD showed significant impairments compared to the control group in sensory processing and modulation, as well as in behavioural and emotional responses as observed in 11 out of 14 sections and 6 out of 9 factors. Differences in all Sensory Profile response patterns were also observed between the two groups of children. Sensory Profile scores showed a moderately negative correlation with CBCL and EACI-P scores in the ADHD group.

**CONCLUSION:**

: These results indicate that children with ADHD may present sensory processing impairments, which may contribute to the inappropriate behavioural and learning responses displayed by children with ADHD. It also suggests the importance of understanding the sensory processing difficulties and its possible contribution to the ADHD symptomatology.

## Introduction

Attention Deficit/Hyperactivity Disorder (ADHD) is a common developmental disorder in childhood with an estimated prevalence of up to 6.4% in school age children^1^. The population affected is rather heterogeneous and shows considerable variation in the degree of symptoms, as well as the frequent presence of associated comorbidities^2^. The DSM-IV-TR^3^ (APA, 2002) has divided ADHD into three subtypes: Predominantly Inattentive subtype (ADHD-I), Predominantly Hyperactive-Impulsive (ADHD-H/I) and Combined Subtype (ADHD-C). In addition to the impairment caused by the core symptoms, researchers and clinicians have suggested that ADHD may also affect children's sensory processing, particularly sensory modulation^4^.

Sensory Processing (SP) is a widely used terminology in the literature to designate a neurological process, and is defined as the ability of the central nervous system to assimilate, process and organize appropriate responses to information. Sensory modulation is the ability to regulate the degree, intensity and nature of a response to a sensory input^5^.

Individuals with sensory modulation difficulties may show behaviour patterns related to decreased or *under responsivity* - poor reactions to relevant stimuli in the environment in the form of passivity, apathy, or lethargy (e.g. they have difficulty knowing where their body is in space, and initiating movements); *sensory seeking - *a constant search for intense stimuli (e.g. they engage in activities that provide more intense sensations for their bodies, they are constantly on the move); and increased or *over responsivity* or exaggerated, aversive or intolerant responses to stimuli (e.g. they are distracted by any stimuli, experience non-harmful stimuli as unpleasant and irritating and thus may exhibit negative, impulsive or aggressive responses)^5^^,^ 6.

These conditions may adversely affect the efficiency of the person's ability to adapt to daily situations, to interact with the environment, to participate in social skills and school activities^6^^-^ 8, and to demonstrate difficulties with attention, emotions^4^^,^ 9 ^,^ 10 and learning^11^.

According to Dunn and Bennett^10^, children with ADHD may not receive and process sensory information properly and consequently, have difficulty producing appropriate adaptive responses at school, at home, and in social settings. This condition may affect motor and functional performance, as well as behavioural aspects of children's lives, including their ability to learn, to organize and to maintain appropriate activity levels^12^. Sensory modulation difficulties among ADHD children have been analyzed in some studies using both behavioural and neurophysiology measures.

Mangeot et al.^9^ reported significantly higher sensory responsivity among ADHD school children than controls, as measured by electrodermal reactivity. Parush et al.^13^ found differences in central processing of somatosensory input among ADHD children with tactile over responsivity, measured by EEG recordings, compared with ADHD children without tactile over responsivity.

From the behavioural point of view, Dunn and Bennett^10^ analyzed the ability of the parent-report questionnaire (Sensory Profile-SP)^14^ to identify and assess children with ADHD. It was reported that they showed significant differences compared to control children on all 14 sections of the Sensory Profile, including their processing of auditory, touch, multisensory, emotional/social responses and behaviour outcomes.

These results were also reported by Yochman et al.^4^ in an Israeli preschooler study. Using the same questionnaire, the authors reported that children with ADHD showed higher sensory responsivity than controls. Cheung and Siu^15^ also reported that Chinese ADHD children showed significantly more sensory processing impairments than children without ADHD disorders. Dove and Dunn^8^ also used the Sensory Profile and reported impaired sensory responsivity and lower scores on Low Registration, Sensation Seeking and Sensation Avoiding patterns in children with learning disorders (both with and without ADHD). Studies using the Short Sensory Profile (SSP)^16^ found that ADHD children's sensory processing was more impaired than that of the controls^6^^,^ 9 ^,^ 16.

Given the multidimensional nature of ADHD, current research has largely focused on cognitive and behavioural abilities related to attentive and executive functions**, **not paying much attention to the role of the sensorimotor dimension. Although few studies in the literature have indicated the presence of SP difficulties in ADHD children, most researchers have worked with a general profile, and few have explored further characterizations of all components of Sensory Processing. More research is needed to explore and characterize SP impairment patterns in ADHD children, and to verify the impact and possible relation between SP difficulties and symptoms presented in their daily-living activities.

From a behavioural point of view, ADHD-C has been reported to compromise adaptive function with higher incidence of interpersonal relationship issues and externalizing behaviour, such as aggressiveness, impulsiveness or oppositional and conduct disorders. In relation to internalizing behaviour, such as anxiety, somatic and other problems, the differences between subtypes tend to decrease^17^. Furthermore, recent research recognizes the importance of self-regulatory mechanisms in determining ADHD symptoms. In addition, the inability to manage and control behaviour, due to inhibitory control difficulties and impaired self-regulation, stimulates the emergence of important emotional symptoms such as low tolerance of disappointment, impatience, anger, anxiety and intense emotional reactions^18^.

Chu and Reynolds^19^ discussed the importance of a multidimensional approach when evaluating and treating ADHD. In this context, since SP impairments are related at the neurological level, affecting sensory-motor, psychological, and behavioural aspects, it could be better studied and identified in children with ADHD. Thus, the present study assessed and compared the sensory responses of children with ADHD and children without this disability. This study also analyzed the possible relationship between SP impairments and behavioural symptoms of children with ADHD.

## Method

### Participants

The sample consisted of 74 children, aged 6-11 (M=8.9, SD=1.49) years, whose parents were the informants. Thirty-seven children with ADHD (30 boys, 7 girls; 24 attending public schools, 13 attending private schools) were recruited from an outpatient clinic, associated with the Universidade Federal de São Paulo (UNIFESP), São Paulo, SP, Brazil, that specialized in the diagnostic of children and adolescents. The children were referred to a multidisciplinary clinical assessment schedule that consisted of psychiatric, neurological and neuropsychological evaluation.

The neuropsychological assessment included the following: the children's intellectual level was tested using the abbreviated (estimated IQ) Wechsler Intelligence Scale for Children (WISC-III)^20^, the attention test using the Conners' Continuous Performance Test (CCPT)^21^, the Automated Working Memory Assessment (AWMA)^22^ test, and the BRIEF (Behaviour Rating Inventory of Executive Functions)^23^ test. The psychiatric interview included criteria based on the DSM-IV-TR^3^, the Child Behaviour Checklist (CBCL)^24^ and the Brazilian version of the Conners Rating Scale - EACIP-P^25^.

The sample was recruited immediately after the diagnosic assessment, prior to the beginning of the medications. Children with pervasive developmental disorders, psychiatric disorders (e.g. bipolar disorders, depressive disorder), neurological disorders (e.g. traumatic and non-traumatic brain injury, such as epilepsy), intellectual disability (IQ<70) and those who were prescribed drugs for ADHD, were excluded.

A DSM-IV-TR-based questionnaire answered by the parents/caregivers found that 21.6% (n=8) of the sample met the criteria for the inattentive subtype (ADHD-I); 19.9% (n=7) for the hyperactive/impulsive subtype (ADHD-H/I); and 59.5% (n=22) for the combined subtype (ADHD-C). The results of the CBCL showed that 13.5% (n=5) presented no comorbidity indicators and 86.4% (n=32) had one or more ADHD-associated comorbidity indicators. Of the 32 children, 40.6% (n=13) met criteria for Affective Disorder indicators; 40.6% (n=13) for Anxiety Disorder indicators; 15.6% (n=5) for Somatic Disorder indicators; 65.6% (n=21) for Opposition Defiant Disorder indicators, and 68.7% (n=22) for Conduct Disorder indicators.

The control group consisted of 37 children paired with the ADHD group by age, gender and type of school (30 boys, 7 girls; 24 at public schools, 13 at private schools). The control group was a convenient sample recruited by the parents/caregivers of the ADHD group by asking classmates and neighbours to participate. We excluded children with hyperactivity and/or inattention indicators, based on the abbreviated Conners Rating Scale (CATRS-10)^25^, and other developmental problems (e.g. convulsions, diseases) based on a health questionnaire answered by their parents.

Sensory processing abilities were assessed using a version of the Sensory Profile^14^ that was translated and adapted for Brazilians^26^. This parent-caregiver report is a measure of the children's responses to daily sensory events and detects behavioural responses that indicate over-responsivity (i.e. low neurological threshold) or under-responsivity (i.e. high neurological threshold).

The questionnaire contained 125 items divided into 14 sections, 9 factors and 4 response patterns. The 14 sections were divided into three categories: 1) Sensory Processing, 2) Modulation and 3) Behavioural and Emotional Responses. The 9 factors - Sensory Seeking, Emotionally Reactive, Low Endurance, Oral Sensory Sensitivity, Inattention/Distractibility, Poor Registration, Sensory Sensitivity, Sedentary, Fine Motor/Perceptual - were based on combined scores from specific items from different sections. The 4 response patterns - Low Registration, Sensation Seeking, Sensory Sensitivity, Sensation Avoiding - were combined scores from specific factors and sections.

The questionnaire used a 5-point Likert scale, corresponding to the frequency of each behaviour (1=Always to 5=Never), where a lower score indicated a higher frequency of undesirable behavioural responses to the sensory events.

Behavioural symptoms of the ADHD children were examined using the Child Behaviour Checklist (CBCL)^24^ and the EACIP-P^25^, a teacher-report questionnaire covering five main areas of behaviour: Hyperactivity/Conduct Problems (EACP-I), Independent Functioning (EACIP-II), Inattention (EACIP-III), Neuroticism/Anxiety (EACIP-IV) and Social Interaction (EACIP-V).

### Procedures

All procedures in this study were approved by the ethics committee of UNIFESP (CEP 1555/09). Informed consent forms were obtained from the children and their parents/caregivers.

The Sensory Profile questionnaire was administered to both groups in a single interview after receiving the written consent of the parents or caregivers. Data for the ADHD group of children were collected at an outpatient unit associated with the UNIFESP, while the control group data were obtained at their homes or schools.

### Data analysis

Since normal distribution was not confirmed for most variables, the non-parametric Mann Whitney test was used to compare the ADHD and control groups' scores, and the Kruskall-Wallis test was used to compare the ADHD-I, ADHD-HI and ADHD-C subtype scores. The magnitude effect (*Cohen d*) was also calculated to determine the strength of the observed differences between variables.

The relationship between SP impairments and behavioural symptoms of children with ADHD was analyzed using Pearson's correlation coefficient. Specifically, the correlation between the Sensory Profile and the CBCL scores, and between the Sensory Profile and the EACI-P scores were analysed with a significance level of p<0.05.

## Results

ADHD children scored significantly lower on most of the Sensory Profile sections, factors and response patterns, suggesting that they may have different patterns of sensory processing and modulation. The greatest amount of difficulty was found to be the adaptive responses to sensory events when compared to typically-developing children.

Significant differences, with moderate to large magnitude effect (p≤0.001, d=0.74 to 2.08), were found between the ADHD and control groups on 11 of the 14 Sensory Profile sections (Table 1).

**Table 1:** Comparison of Sensory Profile section scores among ADHD children and control children.

	Control		ADHD		U	p-value	Cohen d
	M	SD	M	SD			
**Sections**							
**Sensory Processing**							
A. Auditory Processing	31.70	6.11	21.59	5.66	177.00	0.000	1.71
B. Visual Processing	39.78	4.08	33.14	7.59	304.00	0.000	1.08
C. Vestibular Processing	45.95	4.98	37.27	3.85	124.00	0.000	1.95
D. Touch Processing	78.16	7.11	67.43	11.55	302.50	0.000	1.11
E. Multisensory Processing	31.54	8.73	20.30	4.36	49.00	0.000	1.62
F. Oral Processing	47.86	7.95	43.05	10.10	511.50	0.061	0.52
**Sensory Modulation**							
G. Sensory Processing related to endurance/tone	43.24	2.78	39.08	5.30	329.00	0.000	0.98
H. Modulation related to body position and movement	40.70	5.01	34.46	5.48	270.00	0.000	1.18
I. Modulation of movement affecting activity level	21.70	3.23	19.41	4.51	507.00	0.054	0.58
J. Modulation of sensory input affecting emotion responses	15.16	3.57	12.84	2.58	388.50	0.001	0.74
K. Modulation of visual input affecting emotion/activity level	10.32	2.29	9.54	2.36	550.50	0.135	0.33
**Behavioural and Emotional Responses**							
L. Emotional/ Social Responses	68.11	6.98	51.95	8.43	86.50	0.000	2.08
M. Behaviour outcomes Sensory Processing	24.35	3.89	17.35	4.95	197.00	0.000	1.06
N. Items indicating Thresholds for Response	13.57	1.61	10.84	1.80	189.00	0.000	1.59

Significant differences, with moderate to large magnitude effect (p≤0.05, d=0.58 to 2.46), were also observed on 7 of 9 the factors (Table 2). The analysis of response patterns also indicated lower ADHD-group scores for all four response patterns - Low Registration, Sensation Seeking, Sensory Sensitivity and Sensation Avoiding.

**Table 2:** Comparison of Sensory Profile factor and pattern scores among ADHD children and control children

	Control		ADHD				
	M	DP	M	DP	U	p-value	Cohen d
**Factors**							
1. Sensory Seeking	55.24	11.82	36.92	9.78	171.00	0.000	1.68
2. Emotionally Reactive	62.24	7.63	43.11	9.61	68.50	0.000	2.2
3. Low Endurance	43.24	2.78	39.08	5.30	329.00	0.000	0.98
4. Oral Sensory Sensitivity	35.51	6.95	34.11	8.20	624.00	0.512	0.18
5. Inattention/ Distractibility	27.24	5.61	14.68	4.52	76.00	0.000	2.46
6. Poor Registration	33.49	2.99	31.59	3.47	460.00	0.014	0.58
7. Sensory Sensitivity	18.16	2.70	17.49	3.00	603.00	0.355	0.23
8. Sedentary	12.30	3.46	14.14	5.60	500.00	0.045	0.38
9. Fine Motor/Perceptual	13.95	1.75	9.95	3.32	220.50	0.000	1.5
**Patterns**							
Low Registration	119.97	6.78	109.76	11.61	294.00	0.000	1.07
Sensation Seeking	95.95	15.81	71.35	14.56	184.50	0.000	1.61
Sensory Sensitivity	160.49	19.89	130.92	21.68	213.00	0.000	1.42
Sensation Avoiding	98.81	9.83	74.59	13.87	98.00	0.000	2.01

No significant differences were found between ADHD subtypes on the Sensory Profile sections, factors or response patterns, except for the multisensory section (p=0.008, d=1.22), in which ADHD-C (M=18.45, SD=4.25) scored lower than ADHD-I (M=22.88, SD=2.59) and ADHD- HI (M=23.14, SD=3.48).

Pearson's correlation analysis detected a moderately negative correlation (p<0.05, r=-0.34 to -0.49) between the ADHD group's CBCL and Sensory Profile scores. For instance, higher indicators of comorbidity disorders were associated with poorer responses on some sensory processing aspects (Table 3). This correlation was verified with: a) Affective Disorder and auditory processing; visual processing; emotional/social responses; items indicating thresholds for response; Emotionally Reactive; and Low Registration; b) Anxiety Disorder and touch processing; emotional/social responses; and Sensory Sensitivity; c) Attention Disorder and vestibular processing; emotional/social responses; d) Oppositional Defiant Disorder and emotional/social responses; and Emotionally Reactive; e) Conduct Disorder and auditory processing; multisensory processing; emotional/social responses; and Inattention/ Distractibility.

**Table 3:** Correlations between Sensory Profile scores and CBCL scores among children with ADHD.

	Affective		Anxiety		Somatic		Attention		Oppositional Defiant			
	Pearson	p-value	Pearson	p- value	Pearson	p- value	Pearson	p-value	Pearson	p- value	Pearson	p-value
**Sections**												
**Sensory Processing**												
A. Auditory Processing	–0.35	0.031	–0.21	0.213	–0.10	0.569	–0.32	0.057	–0.29	0.084	–0.40	0.014
B. Visual Processing	–0.36	0.029	–0.32	0.051	–0.02	0.887	–0.04	0.825	–0.02	0.928	–0.04	0.803
C. Vestibular Processing	–0.24	0.159	–0.12	0.497	–0.08	0.646	–0.44	0.007	–0.31	0.066	–0.24	0.158
D. Touch Processing	–0.32	0.051	–0.33	0.049	–0.02	0.888	–0.08	0.644	–0.20	0.247	–0.30	0.068
E. Multisensory Processing	–0.16	0.333	–0.19	0.250	–0.17	0.308	–0.24	0.158	–0.31	0.064	–0.36	0.028
F. Oral Processing	0.00	0.990	–0.17	0.328	0.07	0.690	–0.13	0.444	–0.13	0.452	–0.06	0.726
**Sensory Modulation**												
G. Endurance/Tone	–0.27	0.105	–0.10	0.565	–0.20	0.244	–0.25	0.133	–0.10	0.559	–0.17	0.319
H. Position and Movement Modulation	–0.31	0.060	–0.10	0.545	–0.02	0.909	–0.31	0.058	–0.07	0.668	–0.28	0.099
I. Movement affecting Activity Level	0.06	0.721	–0.02	0.926	–0.07	0.699	0.08	0.640	0.12	0.481	–0.07	0.699
J. Sensory affecting Emotion Responses	0.03	0.861	–0.04	0.820	0.21	0.212	0.22	0.184	0.12	0.480	0.15	0.381
K. Visual affecting Emotion/ Activity Level	0.11	0.505	0.13	0.431	–0.05	0.774	0.11	0.529	0.01	0.951	–0.12	0.476
**Behavioural and Emotional Responses**												
L. Emotional/ Social Responses	–0.49	0.002	–0.38	0.019	–0.09	0.596	–0.33	0.044	–0.44	0.007	–0.36	0.029
M. Behaviour outcomes Sensory Processing	–0.04	0.821	0.01	0.964	0.09	0.586	–0.13	0.433	–0.08	0.628	–0.24	0.148
N. Thresholds for Response	–0.33	0.046	–0.07	0.695	–0.03	0.859	–0.09	0.608	0.16	0.330	–0.14	0.394
**Factors**												
1. Sensory Seeking	–0.06	0.706	0.09	0.594	–0.03	0.876	–0.15	0.389	–0.05	0.747	–0.30	0.069
2. Emotionally Reactive	–0.41	0.012	–0.28	0.088	–0.14	0.399	–0.29	0.079	–0.35	0.032	–0.24	0.153
3. Low Endurance	–0.27	0.105	–0.10	0.565	–0.20	0.244	–0.25	0.133	–0.10	0.559	–0.17	0.319
4. Oral Sensory Sensitivity	–0.01	0.945	–0.26	0.128	0.12	0.468	–0.12	0.486	–0.12	0.483	–0.02	0.902
5. Inattention/ Distractibility	–0.29	0.083	–0.18	0.299	0.07	0.696	–0.25	0.135	–0.17	0.315	–0.34	0.042
6. Poor Registration	–0.29	0.077	–0.05	0.778	–0.06	0.741	0.02	0.886	0.12	0.482	–0.05	0.781
7. Sensory Sensitivity	–0.20	0.243	–0.37	0.026	0.07	0.702	–0.31	0.065	–0.19	0.269	0.05	0.765
8. Sedentary	0.08	0.645	–0.01	0.966	–0.14	0.425	0.10	0.545	0.21	0.212	0.05	0.753
9. Fine Motor/Perceptual	–0.21	0.208	–0.04	0.812	0.11	0.529	–0.02	0.895	–0.03	0.837	–0.26	0.127
**Patterns**												
Low Registration	–0.34	0.043	–0.10	0.542	–0.20	0.245	–0.22	0.186	–0.06	0.746	–0.17	0.320
Sensation Seeking	–0.16	0.336	0.02	0.909	–0.02	0.890	–0.22	0.195	–0.07	0.695	–0.31	0.064
Sensory Sensitivity	–0.19	0.272	–0.32	0.057	0.08	0.656	–0.28	0.091	–0.24	0.151	–0.20	0.228
Sensation Avoiding	–0.27	0.112	–0.20	0.241	–0.12	0.477	–0.21	0.215	–0.19	0.260	–0.23	0.168

A moderate significant negative correlation (p<0.05, r=-0.34 to -0.61) was also found between the EACI-P and Sensory Profile scores, suggesting that increased signs of behavioural impairment at school were associated with worse responses on some aspects of the SP (Table 4). This correlation was found between: a) EACI-P I (hyperactivity/conduct problems) and touch processing; and Sensation Avoiding; b) EACI-P II (independent functioning) and behaviour outcomes sensory processing; Fine Motor/Perceptual; c) EACI-P III (inattention) and auditory processing; behaviour outcomes sensory processing; items indicating Thresholds for Response; Inattention/Distractibility, Fine Motor/Perceptual; and Sensation Avoiding; d) EACI-P IV (neuroticism/anxiety) and Thresholds for Response items; e) EACI-P V (socialization problems) and modulation of movement affecting activity level; emotional/social responses; items indicating Threshold for Response; Emotionally Reactive; Sedentary; and Sensation Avoiding items.

**Table 4:** Correlation between ADHD children's Sensory Profile and EACI-P scores.

	EACIP-I		EACIP-II		EACI-PIII		EACIP-IV		EACIP-V	
	Pearson	p-value	Pearson	p-value	Pearson	p-value	Pearson	p-value	Pearson	p- value
**Sections**										
**Sensory Processing**										
A. Auditory Processing	–0.17	0.320	0.19	0.271	–0.38	0.023	–0.20	0.260	–0.33	0.052
B. Visual Processing	–0.17	0.330	0.25	0.150	–0.21	0.237	–0.11	0.532	–0.23	0.186
C. Vestibular Processing	0.03	0.887	0.00	0.984	0.00	0.999	0.33	0.049	0.01	0.966
D. Touch Processing	–0.34	0.046	0.29	0.093	–0.27	0.113	–0.07	0.691	–0.26	0.136
E. Multisensory Processing	–0.31	0.068	0.23	0.179	–0.10	0.550	0.05	0.785	–0.13	0.445
F. Oral Processing	0.01	0.942	–0.05	0.788	0.19	0.269	0.08	0.649	–0.08	0.644
**Sensory Modulation**										
G. Endurance/Tone	–0.07	0.673	0.16	0.370	–0.09	0.623	–0.19	0.275	–0.27	0.119
H. Position and Movement Modulation	–0.29	0.088	0.19	0.285	–0.30	0.078	0.04	0.828	–0.18	0.312
I. Movement affecting Activity Level	–0.22	0.204	–0.12	0.480	–0.23	0.185	–0.04	0.819	–0.46	0.006
J. Sensory affecting Emotion Responses	0.08	0.653	0.00	0.989	0.24	0.171	–0.06	0.724	0.07	0.685
K. Visual affecting Emotion/ Activity Level	0.23	0.178	–0.04	0.822	0.23	0.183	0.22	0.210	0.13	0.443
**Behavioural and Emotional Responses**										
L. Emotional/ Social Responses	–0.22	0.208	0.02	0.910	–0.23	0.183	–0.22	0.214	–0.34	0.047
M. Behaviour outcomes Sensory Processing	–0.23	0.188	0.47	0.004	–0.51	0.002	–0.07	0.704	–0.26	0.137
N. Thresholds for Response	–0.22	0.195	0.31	0.067	–0.38	0.025	–0.40	0.017	–0.34	0.045
**Factors**										
1. Sensory Seeking	–0.29	0.092	0.21	0.218	–0.27	0.115	0.11	0.543	–0.12	0.491
2. Emotionally Reactive	–0.26	0.136	0.04	0.808	–0.27	0.117	–0.25	0.148	–0.46	0.006
3. Low Endurance	–0.07	0.673	0.16	0.370	–0.09	0.623	–0.19	0.275	–0.27	0.119
4. Oral Sensory Sensitivity	0.04	0.831	–0.08	0.645	0.23	0.182	0.04	0.799	–0.04	0.832
5. Inattention/ Distractibility	–0.14	0.410	0.17	0.330	–0.39	0.022	–0.06	0.743	–0.23	0.189
6. Poor Registration	–0.01	0.934	0.01	0.975	–0.02	0.927	–0.11	0.538	–0.11	0.545
7. Sensory Sensitivity	0.02	0.925	0.00	0.999	0.06	0.745	0.21	0.233	0.03	0.875
8. Sedentary	–0.20	0.261	–0.16	0.369	–0.22	0.204	–0.10	0.581	–0.50	0.002
9. Fine Motor/Perceptual	–0.09	0.620	0.56	0.000	–0.42	0.011	–0.14	0.422	–0.10	0.555
**Patterns**										
Low Registration	–0.07	0.689	0.14	0.422	–0.08	0.642	–0.20	0.244	–0.27	0.115
Sensation Seeking	–0.31	0.073	0.22	0.213	–0.30	0.082	0.09	0.611	–0.15	0.401
Sensory Sensitivity	–0.05	0.760	0.03	0.849	0.00	0.985	0.02	0.910	–0.18	0.290
Sensation Avoiding	–0.34	0.047	0.12	0.484	–0.45	0.007	–0.24	0.168	–0.61	0.000

## Discussion

Sensory Profile abilities of ADHD children were assessed according to their response to daily sensory events. In addition, the possible relationship between sensory processing impairments and behavioural symptoms presented by ADHD children was also analyzed.

Our results indicated significant differences on 11 of the Sensory Profile's 14 sections, on which ADHD children scored lower. These results are consistent with those reported by authors who have used the same instrument. Dunn and Bennett^10^ found significant differences in all 14 sections, suggesting that ADHD children had more sensory processing impairments than their control group. However, they only analyzed Sensory Profile sections and many of their ADHD children were under medication. Yochman et al.^4^ also found differences in 11 sections and worse ADHD group responses, except for vestibular processing, tone/endurance, and emotional response.

Others authors have also reported similar findings to our own, such as significant differences between ADHD and control groups for auditory, visual, touch and oral processing, indicating that ADHD children may have sensory processing difficulties related to these systems^4^^,^ 9 ^,^ 13. In our study, however, there was only a significant difference between groups for the oral processing system.

In regard to Sensory Profile factors, we found significant differences between ADHD and the control group scores in 7 out of 9 factors, the exceptions being oral sensitivity and sensory sensitivity. Yochman et al.^4^ also found significant differences in 6 out of 9 factors with ADHD children scoring lower except for Low Endurance, Poor Registration and Sensory Sensitivity. However, their sample consisted only of preschoolers aged 4-6. Several functions are still being developed at this age and some symptoms may yet change as the brain develops.

Our study also found that ADHD children experienced major difficulties showing significance in all four Sensory Profile response patterns: Sensation Avoiding, Sensory Seeking, Sensory Sensitivity and Poor Registration. This dimension was previously analyzed only by Dove and Dunn^8^, who compared typically developing and specific learning disability children (the latter, with and without ADHD) and found that the clinical group obtained low Sensory Profile scores for Sensory Seeking, Sensation Avoiding and Poor Registration. However, there was no specific comparison between the ADHD children and controls.

Cheung and Siu^15^ specifically analyzed scores on each Sensory Profile item and found that the ADHD group scored lower than the controls. However, since they did not analyze the scores obtained for sections, factors and response patterns, these dimensions could not be compared with our results.

This study found that ADHD children had significant Sensory Processing impairments on dimensions such as emotional/social responses (section L) or Emotional Reactivity (factor 2), containing items related to self-esteem, frustration tolerance, irritability, anxiety and other emotional aspects. Some authors suggest that these behaviours may be associated with ADHD children's executive function deficits, impeding adequate performance of daily tasks and social skills^27^; but may also be the result of inadequate sensory modulation of sensory system inputs^14^.

Sensory processing impairments were also observed on dimensions such as vestibular processing (section D), modulation of body position and movement (section G) and Sensory Seeking (factor 1), particularly for items concerning under-responsivity to vestibular and proprioceptive systems, showing excessive body movement and continuous stimulus seeking. These results pose the question of whether ADHD symptoms, such as constantly seeking body movement and stimuli, as described by the DSM-IV^23^ and explained by inhibitory control deficits, may not also be influenced by the children seeking vestibular and proprioceptive sensory stimuli as a behavioural response to these children's high thresholds for these systems.

Our results also showed impairment on auditory-processing items (section A), which reflect overly responsive behaviours but also under-responsivity. It is important to consider that some of the issues regarding Sensory Profile auditory processing are already described in DSM-IV (e.g. distracted or has trouble functioning if there is a lot of noise around). However, assessment of auditory processing can help in understanding the basis of the behaviour of distractibility. Furthermore, our results suggest that a low threshold for sensory stimuli could contribute to distractibility in relation to an auditory stimulus in some ADHD children, whereas a high threshold could contribute to inattentive behaviour in others.

Therefore, from the Sensory modulation perspective, inattention could be present in individuals with under-responsivity (i.e. high threshold) who require more intense stimuli. Distractibility could be present both in under-responsive individuals who tend to seek stimuli in order to be organized, and in over-responsive (i.e. low threshold) individuals, who respond to all stimuli, with both types presenting higher activity levels.

Significant impairments were also observed in all four-response patterns. According to Dove and Dunn^8^, each response pattern may have different repercussions for learning. In the presence of Sensory Seeking, the individual may seek movements and constant stimuli to obtain more sensory input (e.g. does not sit still, moves a lot on the seat). In the presence of Sensation Avoiding, the individual displays the need to avoid and aversion to sensory experiences (e.g. is disturbed by noise in the class whenever others bump into his/her desk). Whenever there is Poor Registration, the individual tends to respond slowly to the stimuli (e.g. does not retain information given by teachers, does not apprehend details in order to complete the required tasks). Lastly, in the presence of Stimuli Sensitivity, the individual easily responds to any stimuli (e.g. does not concentrate on the proposed task, does not finish what he/she has started, being distracted by other stimuli).

CBCL and EACI-P scores showed moderate negative correlation with Sensory Profile scores, suggesting that the increased presence of behavioural-symptom indicators were associated with worse responses for some aspects of the Sensory Processing. This correlation was found between: Auditory processing and Affective Disorder, Conduct Disorder (CBCL), and inattention (EACI-P); Vestibular processing and Attention Disorder (CBCL); Multisensory processing and Conduct Disorder (CBCL); Fine Motor/Perceptual and independent functioning and inattention (EACI-P); touch processing and Sensory Sensitivity, and Anxiety Disorder (CBCL) and hyperactivity/conduct problems (EACI-P); Sensation Avoiding and hyperactivity/conduct problems, inattention, socialization problems (EACI-P); Thresholds for Response and neuroticism/anxiety and socialization problems (EACI-P); and, Modulation of movement affecting activity level and socialization problems (EACI-P).

Mangeot et al.^9^ also found a higher correlation between the Short Sensory Profile's Tactile Sensitivity and the CBCL's Aggressive Behaviour and Somatic Complaints items. The relationship between sensory over-responsivity and anxiety was also analyzed by Reynolds and Lane^28^, who found that ADHD children with over-responsivity were more susceptible to show anxiety than children without over-responsivity or control children.

According to Roberts et al.^29^, different abilities and expression of behaviours relate to the individual's self-regulation, which refers to one's ability to regulate responses to specific stimuli, involving physiological, emotional and behavioural factors, and their interdependencies. Therefore, the ability to process sensory information is one of the factors that may influence individual differences in terms of self-regulation.

From the Sensory Modulation perspective, there is an interaction between the external dimension corresponding to culture, environment, relationships and tasks, and the internal dimension, which includes sensation, emotion and attention^30^. Thus, behaviour is generated based on an adequate interaction of such dimensions, so the presence of sensory modulation difficulties could cause emotional states including depression, anxiety, fear, aggressiveness and emotional lability^14^^,^ 30, in addition to attentional states such as distractibility, impulsiveness and hyperactivity^9^^,^ 30.

Our findings did not indicate significant differences between ADHD subtypes on Sensory Profile scores, except for multisensory processing (section E). Engel-Yeger and Ziv-On^6^ compared Sensory Processing between ADHD subtypes using the abbreviated version - Short Sensory Profile^16^ - and also found no significant differences between groups. As in the case of our own study, differences might not have been found due to the small number of subjects in each ADHD subtype group, as well as the concomitance of several comorbidities associated with ADHD, thus impeding a more specific analysis of Sensory Processing in ADHD subjects.

## Conclusion and limitations

Previous studies^4^^,^ 6 ^,^ 9 ^,^ 10 ^,^ 15 have suggested that ADHD children's Sensory Processing and Modulation patterns are significantly different to those of typically developing children. Our results reproduce previous findings while extending comprehension of this pattern in ADHD, since i) our sample members were not on medications, so our Sensory Processing analysis was free of the effects of medication; ii) the sample age range was broader; iii) SP scale sections, factors and response patterns were analyzed, and iv) impairment of SP abilities in ADHD was discussed.

Furthermore, the present study's findings suggest that ADHD children may have sensory modulation impairments which may contribute to behaviour and learning inappropriate responses displayed by children with ADHD, suggesting the importance of considering and studying SP difficulties and the possible contribution to the symptomatology of ADHD. In clinical practice, this discussion is relevant because it suggests the possibility of considering and including sensory strategies and resources when treating the symptoms of children with ADHD.

Our results should be interpreted in light of certain limitations, since the small number of ADHD subtype subjects prevented effective comparison of their sensory-processing abilities. Future research requires a larger sample to investigate sensory modulation differences between ADHD subtypes. Another limitation was the extent of comorbidities in ADHD children hindering more specific SP analysis. It also might be of interest to analyze the degree to which the sensory processing symptoms improve when affected by medication.
